# Liquid Biopsy in Gastrointestinal Cancers

**DOI:** 10.3390/diagnostics8040075

**Published:** 2018-10-29

**Authors:** Aman Saini, Yash Pershad, Hassan Albadawi, Malia Kuo, Sadeer Alzubaidi, Sailendra Naidu, M-Grace Knuttinen, Rahmi Oklu

**Affiliations:** Division of Vascular and Interventional Radiology, Laboratory for Minimally Invasive Therapeutics, Mayo Clinic, Phoenix, AZ 85054, USA; saini.aman@mayo.edu (A.S.); ypershad@stanford.edu (Y.P.); albadawi.hassan@mayo.edu (H.A.); malia1274@gmail.com (M.K.); alzubaidi.sadeer@mayo.edu (S.A.); naidu.sailen@mayo.edu (S.N.); knuttinen.grace@mayo.edu (M.-G.K.)

**Keywords:** liquid biopsy, circulating tumor cells, circulating tumor DNA, gastrointestinal cancer

## Abstract

Liquid biopsy is the sampling of any biological fluid in an effort to enrich and analyze a tumor’s genetic material. Peripheral blood remains the most studied liquid biopsy material, with circulating tumor cells (CTC’s) and circulating tumor DNA (ctDNA) allowing the examination and longitudinal monitoring of a tumors genetic landscape. With applications in cancer screening, prognostic stratification, therapy selection and disease surveillance, liquid biopsy represents an exciting new paradigm in the field of cancer diagnostics and offers a less invasive and more comprehensive alternative to conventional tissue biopsy. Here, we examine liquid biopsies in gastrointestinal cancers, specifically colorectal, gastric, and pancreatic cancers, with an emphasis on applications in diagnostics, prognostics and therapeutics.

## 1. Introduction

Globally, gastrointestinal (GI) cancers contribute significantly to cancer related mortality. In 2015, there were 1.7 million cases of colorectal cancer (CRC) and 832,000 cancer-related deaths [1], ranking it second in terms of cancer-related deaths worldwide. Gastric cancer ranked fifth for cancer incidence (1.3 million) and third for cancer deaths (819,000) [1]; other GI cancers also contribute significantly to cancer-related mortality including pancreatic cancer which often presents at advanced stages and has short median survival. The high mortality of GI cancers has prompted advancements in the field of diagnostics with the goal of early diagnosis, which can lead to improved patient outcomes and survival. 

Tissue biopsy remains the gold standard for the establishment of a cancer diagnosis. This allows for tumor classification, assessment of invasiveness and degree of differentiation, characterization of genetic makeup and mutational status, and provides insight into prognosis; such information can help guide and personalize treatment strategy. However, tissue biopsies are invasive, costly, and not without complications; sometimes, tissue is not amenable to biopsy due to anatomic location or an underlying coagulopathy. Moreover, tissue biopsies only offer a snapshot of a tumor at one particular time-point and may not always reflect the complete genetic makeup of a neoplasm which is undergoing constant evolution. Intratumoral heterogeneity, clonal evolution, and selection often lead to therapy resistance; serial biopsies to reassess the genetic landscape within a malignancy are not practical due to the potential complications of the procedure. 

**Figure 1:**
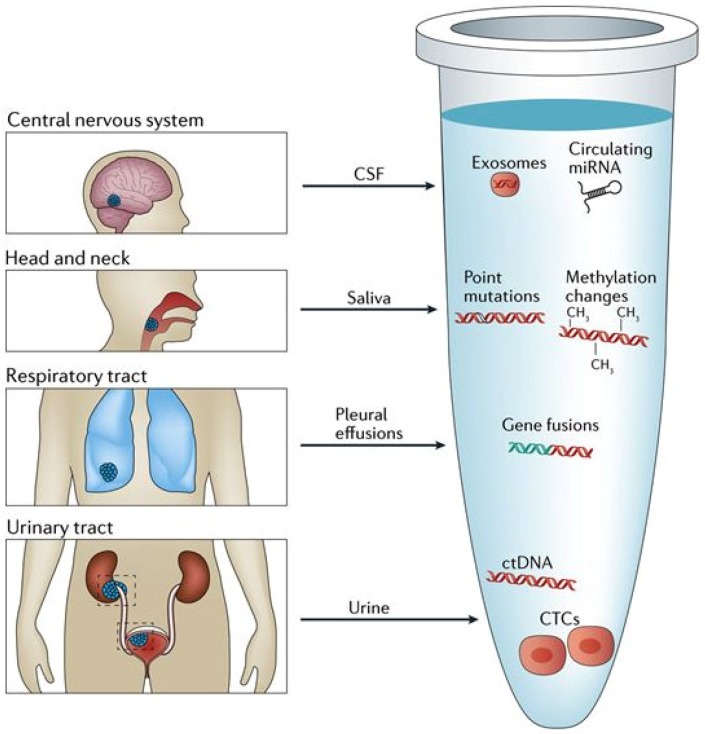
Sources of liquid biopsy material. A variety of biologic fluids can contain tumor-derived genetic material. In addition to blood, these fluids can include cerebrospinal fluid (CSF), saliva, pleural effusions, and urine. ctDNA and CTC’s can be extracted from this fluid and genetic alterations including point mutations, methylation changes, and gene fusions can be analyzed. Reproduced with permission from [2]; published by Springer Nature Limited, 2017.

Liquid biopsy represents an alternative, less-invasive, and more comprehensive approach to the diagnosis of cancer, characterization of tumors, personalizing of treatments and monitoring of treatment response. A liquid biopsy is any sample of biologic fluids such as blood, saliva, cerebrospinal fluid, or urine that may contain tumor genetic material (Figure 1). Peripheral blood samples as liquid biopsy material have been widely investigated; enrichment and analysis of circulating tumor cells (CTCs), circulating tumor DNA (ctDNA), or other sources of a tumors genetic material (exosomes, RNA, proteins etc.) from peripheral blood is an area of active research. Although a variety of these genetic markers have been investigated in GI cancers, many studies frequently focus on either CTCs or ctDNA, and therefore we will focus primarily on these markers and their roles in colorectal, gastric and pancreatic cancers.

### 1.1. Circulating Tumor Cells

**Figure 2:**
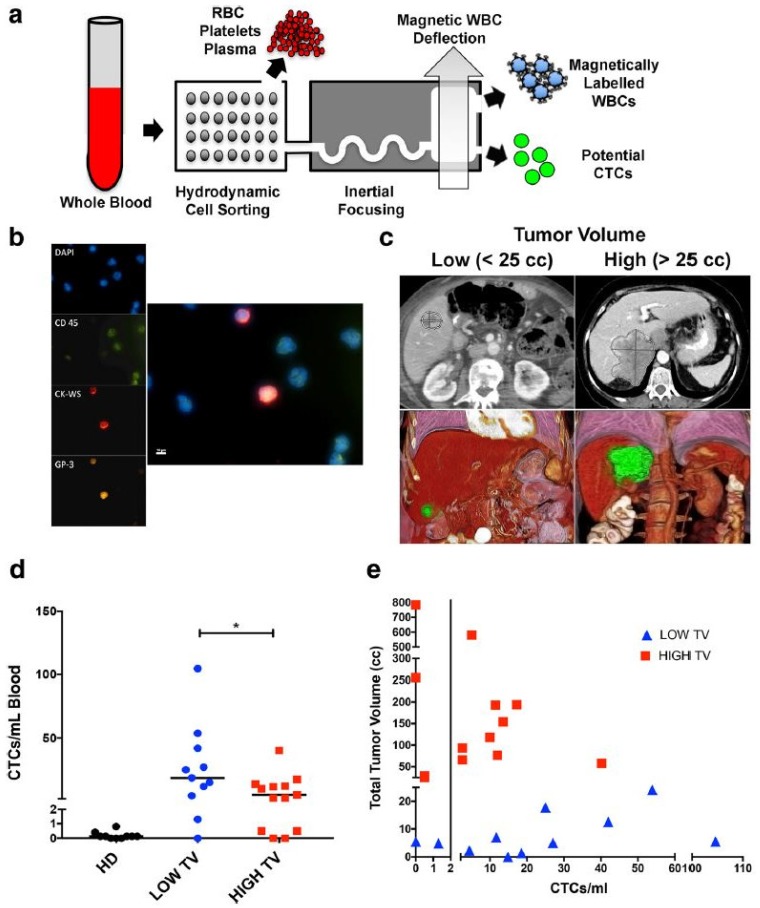
Detection of CTC’s using microfluidics. (**a**) Diagram of CTC isolation using microfluidic chips. (**b**) Immunofluorescent staining of CTC’s. (**c**) CT scan of liver (top) with 3D reconstruction (bottom) demonstrating high and low tumor volume (TV) (**d**) CTC’s/mL of blood in healthy donors (HD) versus patients with high and low TV. Bar = median. * *p* = 0.045. (**e**) Scatter plot demonstrating tumor volume (cc) versus CTC’s/mL. Spearman *r* = 0.3043. Reproduced under open access [16].

Circulating tumor cells are whole cells that detach from a primary or metastatic tumor and then circulate through the bloodstream. Whether this detachment is an active or passive process is unclear, with some hypothesizing that tumor cells undergo an “epithelial to mesenchymal transition” before detaching [3], while others believe that cells simply break off in clusters. Although certain aggressive tumors have been noted to release large amounts of CTC’s into circulation daily [4], oligometastases are more often observed clinically, highlighting the potential inefficiency of the metastatic process [5]. Though CTC’s are highly diluted within the blood [6,7,8], they can be detected by their physical (e.g., size, density, and electric charge) or biological (cell surface protein or gene expression) properties [9]. Investigators have used a number of enrichment techniques with varying results. Reverse transcription polymerase chain reaction (RT-PCR) has been used in a number of studies [10,11] to detect various gene target markers with high sensitivity including carcino-embryonic antigen (CEA), cytokeratin (CK) 19, CK20, and CD133 [12]. Epithelial cell adhesion molecule (EpCAM) is a commonly used marker for CTC enrichment and a FDA-approved immunomagnetic assay called Cell-Search^®^ is readily available for this purpose. A number of studies examining the role of CTC’s in GI cancers have used Cell-Search^®^ for CTC detection with good results [13,14]. The use of microfluidic chips for CTC isolation using membrane epitope independent methods is also a rapidly developing field, with preliminary results showing great promise [15,16] (Figure 2). Nonetheless, these enrichment methods have achieved variable outcomes, even when examining similar cancer types. Therefore, further refinement and standardization are needed prior to clinical implementation.

### 1.2. Circulating Tumor DNA

Circulating tumor DNA (ctDNA) consists of small (70–200 base pair) fragments of tumor DNA circulating freely alongside the DNA of normal, healthy cells within the blood. The physiologic presence of circulating nucleic acids within the serum was first reported by Mandel and Metais in 1948 [17], however the properties of these DNA fragments were not elucidated until more recently. Circulating tumor DNA is released from primary, metastatic, and circulating tumor cells undergoing apoptosis or necrosis, and has also been found in exosomes [18]. The half-life of ctDNA is short, ranging from 15 min to 2.5 h and these fragments are cleared primarily by the liver and kidney [19]. ctDNA is being constantly released into the blood stream with blood concentration levels often proportional to disease burden [20]. Moreover, several studies have demonstrated high concordance between genetic alterations within solid tumors and those within ctDNA [11,21,22], helping to differentiate ctDNA from the circulating DNA associated with normal cell turnover.

**Figure 3:**
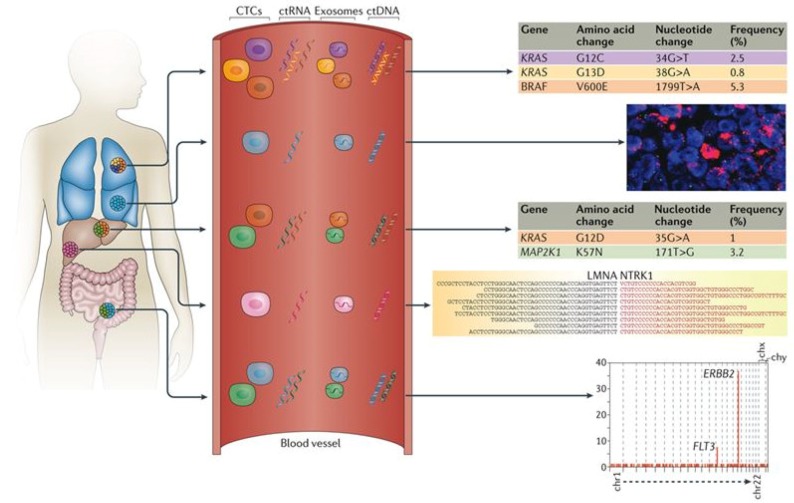
Liquid biopsy can non-invasively assess the molecular heterogeneity of cancers. CTC’s, ctDNA, ctRNA, and exosomes can be used to characterize the heterogeneity of distinct tumor lesions with different genetic mutations or alterations. The tables demonstrate the detection of point mutations in various genes through candidate-gene or next-generation sequencing analysis. The fluorescence micrograph demonstrates the use of fluorescence *in situ* hybridization analysis to detect copy-number variations. Reproduced with permission from [2]; published by Springer Nature Limited, 2017.

The quantity of ctDNA in blood is likely influenced by tumor burden. Once extracted, ctDNA can be analyzed for previously characterized or highly recurrent mutations (e.g., KRAS), or for new genetic alterations (hyper-/hypo-methylation, chromosomal, copy number changes or point mutations) [23] (Figure 3). Highly sensitive methods for detecting these genetic alterations are critical and a number of specific techniques exist for doing so. Methylation-specific quantitative PCR (qPCR) has proven its utility in CRC ctDNA analysis [24], as has low pass sequencing for detecting large somatic copy number variations [25]. Moreover, advances in next-generation sequencing have allowed for the detection of multiple point mutations across many genes [26], further enhancing ctDNA detection. 

## 2. Liquid Biopsy as a Diagnostic Tool

The use of liquid biopsy, and in particular ctDNA, as a diagnostic tool to aid in screening/early detection, disease monitoring, assessment of residual disease, and disease recurrence in GI cancers is a rapidly growing area of research. Perhaps the biggest challenge to date for the clinical implementation of ctDNA as part of screening exams has been the lack of sensitivity and specificity of ctDNA tests in patients with early-stage disease, when the amount of ctDNA in the plasma can be <1 mutant template molecule per milliliter of plasma [20]. Progress has been made, however, in the detection and isolation of ctDNA [20] for a number of localized and metastatic cancers, including CRC. More recently, Molparia et al. report a pilot study to detect large scale somatic copy number variants (CNV’s) in early stage CRC, which have been shown to contribute more molecules to ctDNA signal when compared to point mutations. With a cohort of 25 CRC and 25 healthy patients, they achieved 100% specificity and 79% sensitivity in discriminating between CNV’s from CRC patients and the healthy controls [25]. The methylation status of various genes has also been recognized as a biomarker of colonic neoplasia although the detection of such genes with conventional assays has been difficult. However, novel methylation assays have been developed and demonstrate synergistic effects when combined with fecal immunochemical tests, resulting in increased diagnostic accuracy for the early detection of colorectal cancer [27]. These results allude to the possibilities of liquid biopsy as an adjunct, and eventual alternative, to current CRC screening strategies.

A recent meta-analysis of 16 studies including a total of 1193 patients was performed to assess the diagnostic value of ctDNA in gastric cancer [28]. The researchers demonstrated a pooled sensitivity and specificity of 62% and 95% respectively, while also showing the presence of certain ctDNA markers to be correlated with adverse clinicopathologic features such as larger tumor size or advanced stage. Within the meta-analysis, significant heterogeneity, potentially from ctDNA detection method, gene target, and patient race, was observed highlighting the need for more consistent methodology and experiment design in order to fully clarify the diagnostic role of ctDNA in gastric cancer [28]. In localized pancreatic cancer, ctDNA has been detected in up to 43% of patients at the time of diagnosis [29], and more recently, analyses of genetic material from pancreatic tumor-derived exosomes have shown great promise at earlier detection [18,30]. Melo and colleagues isolated pancreatic tumor-derived exosomes using a cell surface proteoglycan, glypican 1, which is specifically enriched on pancreatic cancer exosomes. Detection of these exosomes distinguished healthy subjects and those with benign pancreatic disease from patients with early- and late-stage pancreatic cancer with 100% sensitivity and specificity [18]. Moreover, these results outperformed the current standard pancreatic cancer biomarker CA 19-9, supporting the utility of these exosomes as biomarkers, even in early disease. 

In summary, liquid biopsy for early cancer detection is a rapidly evolving field that shows great promise and certain blood tests have already been approved by the FDA [31] or are being developed [32]. CancerSEEK is a single, multianalyte blood test that is able to detect 8 localized cancers (i.e., ovarian, liver, gastric, pancreatic, esophageal, colorectal, breast and lung) with varying sensitivities and specificities [32]. Its assay combines tests for ctDNA and protein biomarkers to enhance the diagnostic accuracy and aid in localizing specific tumors. Although more prospective studies of this test in larger populations are necessary, initial results appear promising for cancer screening. 

The use of ctDNA for monitoring of residual disease and recurrence has also been studied. For patients with early-stage GI cancers, curative surgery remains a cornerstone of treatment. In CRC, ctDNA has proven useful as a surrogate marker of minimal residual disease after curative surgery and may offer improvements over conventional diagnostic imaging in assessing residual disease [33,34]. Enhanced detection of residual disease in these patients may help to better select patients for adjuvant treatments including chemotherapy and/or radiotherapy. Liquid biopsy as a tool for monitoring disease recurrence has also been well studied and the use of serial profiling has allowed for detection of recurrence up to 6 months before radiological imaging in CRC and pancreatic cancer patients [29,35,36]. For metastatic CRC, recent studies [34,35,37] demonstrate a decline and subsequent rise in ctDNA levels in response to chemotherapy and acquisition of resistance respectively, indicating that longitudinal monitoring of ctDNA may provide an alternative to the measurement of blood tumor markers, which often lack sensitivity and specificity [38]. Investigations on ctDNA for pancreatic cancer show similar findings, with changes in ctDNA corresponding closely with radiological imaging, CA19-9 levels, and recurrence [29,36]. 

## 3. Liquid Biopsy as a Prognostic Tool

Through identifying patients at high risk of recurrence, giving insight into disease progression and survival, and correlating with changes in tumor burden, liquid biopsies have demonstrated great value as a prognostic tool in GI cancers [23,36,39,40,41]. In a large study of 735 Duke’s stage B and C CRC patients who underwent curative surgery, CTC’s were detectable in nearly 25% of postoperative patients and their presence was associated with significantly shorter disease-free survival (DFS) and overall survival (OS) [12]. In a study by Phillip et al. of 311 CRC patients, the prognostic significance of methylated HLTF and HPP1 was examined and confirmed using methylation-specific qPCR [24]. Investigators discovered that detection of methylated HLTF and HPP1 in the serum of pre-therapeutic CRC patients was associated with adverse clinicopathologic features, metastatic disease and shortened OS. These findings mirror those of Tham et al. [42], who demonstrated the prognostic significance of methylated TAC1 and SEPT9 in postoperative Stage I-III CRC patients. Increased serum methylation of TAC1 at 6 months, and SEPT9 at one year follow up, were independent predictors of recurrence and cancer-specific survival. Interestingly, detection of these two markers at their respective follow up times was more strongly associated with recurrence as compared to CEA levels. More recently, Yao et al. noted RAS/BRAF mutation status in ctDNA as a prognosticator of poor progression free survival (PFS) in metastatic CRC patients treated with first line therapy, as has been well characterized in tumor genomic analysis [43,44]. These results reflect those of other studies examining the prognostic significance of detectable mutations in CRC ctDNA [35,37,43,45]. Circulating tumor DNA in CRC has also been shown to be effective in monitoring disease recurrence after completion of all treatments, further exemplifying its prognostic value. In a recent study comparing a novel two gene blood test to CEA for the detection of recurrent CRC for patients in remission after undergoing primary treatment, the novel ctDNA blood test was found to be more sensitive for recurrence versus CEA and the odds of recurrence given a positive blood test were twice as high when compared to CEA [46]. 

Liquid biopsy as a prognosticator in gastric cancers has been widely studied in CTC’s, with multiple studies associating their presence with decreased PFS and OS [10,13,47,48,49]. Uenosono et al. [13], in a study comprised of 251 patients split into resectable and non-resectable groups, found that CTC presence before and after surgery in the resection group was associated with lower DFS and OS. These results were similar for the non-resectable group, signifying the presence of CTC’s as an independent prognostic factor determining OS in GC patients. A 2014 meta-analysis of 19 studies by Wang et al. significantly associated pre-operative CTC positivity with poor DFS, and OS. Furthermore, their presence was also associated with regional lymph node metastasis, vascular invasion, depth of infiltration, and advanced clinical stage [10]. A meta-analysis by Zhang et al. mirrored similar findings [47]. Another prognostic indicator that has been of interest to gastric cancer researchers is Survivin, a known inhibitor of apoptosis. In gastric cancer liquid biopsies, Survivin expression has been shown to independently predict poorer survival [49,50,51]. Cao et al., in a study of 98 gastric cancer patients who underwent curative resection, demonstrated that postoperative Survivin mRNA expression was associated with increased tumor size, nodal metastasis, and high grade [49]. A 2013 meta-analysis by Liu et al. [51], consisting of 16 studies and 1365 patients, echoed the negative impact of Survivin expression on survival. Interestingly, only cytoplasmic expression of Survivin had a significant impact on overall survival, whereas expression in the nucleus had no effect on prognosis. 

In patients with pancreatic cancer, the presence of ctDNA at diagnosis, and after treatment, portends a poor prognosis [21,52,53]. From a study published in 2017, which included 135 patients with resectable, locally advanced, or metastatic pancreatic cancer, the presence of detectable ctDNA in peripheral blood samples was strongly correlated with poorer survival when compared to those with no detectable ctDNA (19.0 vs. 6.5 months; *p* < 0.001) [52]. In patients who underwent resections, the detection of ctDNA after surgery was associated with shorter DFS and OS. Positivity for KRAS mutations in ctDNA has also been associated with poor prognosis in various pancreatic cancer studies [22,44,54]. A meta-analysis [54] of 623 patients demonstrated significantly worse PFS and OS in those with detectable CTC’s, and a similar negative influence on survival was shown for patients with detectable ctDNA positive for KRAS mutations. When considered collectively, these studies demonstrate various clinical biomarkers that can be used to predict the prognosis of gastrointestinal cancers.

## 4. Liquid Biopsy as a Therapeutic Response Tool

**Figure 4:**
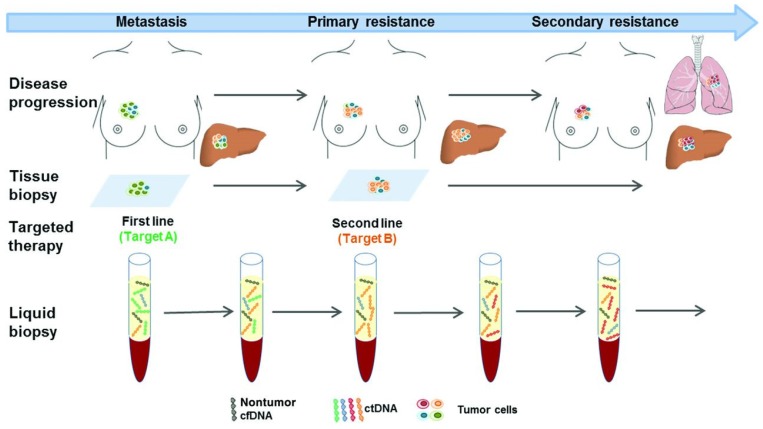
Serial liquid biopsies to monitor therapeutic response and treatment resistance. Hypothetical diagram of a patient with metastatic breast cancer. First- and second-line treatments are based upon tissue biopsy which does not completely assess the heterogeneity of metastatic disease, nor does it account for treatment resistance over time. Serial liquid biopsies could help to characterize tumor heterogeneity, detect treatment resistance and disease progression before it becomes apparent clinically. Reproduced with permission from [58].

Another application of liquid biopsy involves its ability to identify therapeutic targets, predict treatment response, and assess the evolution of tumor resistance over time (Figure 4). Although the field is still in its infancy with respect to these uses, early studies show great potential in these areas, particularly with respect to CRC. In patients with metastatic CRC, the number of CTC’s detected before and during chemotherapy have been shown to independently predict PFS and OS, and have been associated with failure of therapy [55]. More recently, Garlan et al., in a prospective cohort of 82 metastatic CRC patients, demonstrated that changes in ctDNA concentration could predict the efficacy of first- or second-line chemotherapy [56]. In this study, plasma ctDNA samples were collected before the first, second and/or third cycle of chemotherapy in an attempt to discover various gene mutations (KRAS, BRAF, TP53) or hypermethylation status (WIF1, NPY). Patients were then classified into “good” or “bad”ctDNA responders based upon the evolution of ctDNA concentration. Those in the “good” response group had a significantly better objective response rate and longer median PFS (8.5 vs. 2.4 months; *p* < 0.001), suggesting the use of ctDNA concentration as a marker of therapeutic efficacy [56,57].

Clonal evolution and treatment resistance patterns in CRC, particularly to epidermal growth factor receptor (EGFR) blockade, have been well studied, with ctDNA showing a high sensitivity for detecting resistance associated mutations in KRAS, BRAF, and EGFR [20,59,60,61]. Siravegna et al. recently used ctDNA to track clonal evolution during treatment with EGFR-specific antibodies cetuximab and panitumumab. They noted emergence of KRAS mutated clones (and thus disease progression) following treatment with these anti-EGFR antibodies, followed by a subsequent decline in these KRAS mutated clonal populations after removal of EGFR blockade, highlighting the dynamic nature of these cell populations. Moreover, these results suggest the suitability of EGFR re-challenge after salvage chemotherapy—an idea whose clinical efficacy was also confirmed in this study [59]. Clearly, the clinical utility of longitudinal monitoring of ctDNA to alter and adapt treatment strategies shows great potential. 

In advanced GC patients, a recent, prospective, 136 patient study examined CTC levels prior to, and 6 weeks after, the first cycle of chemotherapy. Those patients with an unfavorable CTC level (≥3 CTC per 7.5 mL) after 6 weeks of treatment had significantly poorer objective response rates, PFS and OS [62]. These results were similar to a previous study [14] and suggest, along with the previously mentioned data, that the monitoring of liquid biopsy biomarkers may be an effective way of rapidly identifying ineffective treatments. In a group of 168 patients with diverse cancers, Schwaederle and colleagues were able to identify potentially actionable genetic alterations in 42% of patients. Approximately 75% of these patients had alterations that were actionable by an already approved FDA drug (off-label use), while a smaller percentage (12%) had actionable alterations treatable by a drug approved for their disease (on-label) [26], emphasizing the future clinical utility of liquid biopsy in cancer patients.

## 5. Conclusions

Liquid biopsy and its potential clinical utility, particularly in gastrointestinal cancers, is well studied and shows great potential. However, a considerable amount of progress needs to be made prior to the widespread clinical implementation of liquid biopsy in cancer care. Specifically, additional prospective, multi-institution studies must validate preliminary findings for a wide range of cancers. Clear, standardized, and reproducible procedures for enhancing and detecting minute concentrations of molecules will be necessary. Moreover, further development of clinically implementable technologies such as cost-effective and sensitive assays for detecting and analyzing genetic alterations are needed. Finally, improving patient selection and timing of liquid biopsy necessitates advances in knowledge of tumor biology and mechanisms of treatment resistance. Nonetheless, liquid biopsy has the potential to significantly impact cancer diagnostics and treatment paradigms.
